# Intergenerational violence in Burundi: Experienced childhood maltreatment increases the risk of abusive child rearing and intimate partner violence

**DOI:** 10.3402/ejpt.v6.26995

**Published:** 2015-12-15

**Authors:** Anselm Crombach, Manassé Bambonyé

**Affiliations:** 1 Department of Psychology, University of Konstanz, Konstanz, Germany; 2 Department of Clinical Psychology, University Lumière of Bujumbura, Bujumbura, Burundi; 3 NGO Vivo International e.V., Konstanz, Germany

**Keywords:** Childhood maltreatment, intimate partner violence, intimate partner intimidation, cycle of violence, Burundi

## Abstract

**Background:**

Experiencing abuse during childhood affects the psychological well-being of individuals throughout their lives and may even influence their offspring by enhancing the likelihood of an intergenerational transmission of violence. Understanding the effects of childhood maltreatment on child-rearing practices and intimate partner violence might be of particular importance to overcome the consequences of violent conflicts in African societies.

**Objective:**

Using Burundi as an example, we aimed to explore the associations between childhood maltreatment, intimate partner violence, perceived partner intimidation, gender and the probability of violently acting out against one's own children or romantic partner.

**Methods:**

Amongst a sample of 141 men and 141 women in the capital of Burundi, we identified those who had biological children and those who lived or had lived in relationships. Using culturally appropriate instruments, we enquired about their exposure to childhood maltreatment and partner violence as well as their inclinations to act out violently.

**Results:**

We found that childhood maltreatment and perceived partner intimidation were strong predictors for the perpetration of violence against children. Moreover, we found that women were more likely to use violence against children if they experienced partner violence and less likely to resort to violence if they felt intimidated. Men were more likely to perpetrate violence against their partner. Childhood maltreatment was again a strong predictor. The more women experienced partner violence, the more they fought back.

**Conclusions:**

Childhood maltreatment is a strong predictor for domestic violence and has to be addressed to interrupt the cycle of violence in post-conflict countries.

Intergenerational violence—that is, passing on aggressive behavior to the next generation—is becoming recognized as an essential factor working against the psychological well-being and social functioning of descendants. Experienced childhood maltreatment enhances the risk for mental disorders (Teicher & Samson, 2013) as well as the likelihood of future violent offenses and domestic violence (e.g., Kwong, Bartholomew, Henderson, & Trinke, 2003; Weaver, Borkowski, & Whitman, 2008). Initial research on the genetic and epigenetic levels indicates that the effects of childhood maltreatment may even affect our biological inheritance. Several studies observed alterations in the expression of genes related to the stress sensitivity of the hypothalamus pituitary adrenal axis in individuals who experienced childhood maltreatment or whose mothers experienced intimate partner violence during pregnancy. Abusive environments seem to mediate these alterations, which might transcend several subsequent generations. The resulting increased sensitivity to stress and the associated emotional dysregulation are considered as substantial factors explaining the elevated vulnerability to develop mental disorders and might also contribute to aggressive behavioral patterns in individuals with histories of childhood maltreatment (Ramo-Fernandez, Schneider, Wilker, & Kolassa, 2015; Romens, McDonald, Svaren, & Pollak, 2014). However, the majority of the studies assessing risk factors and consequences of childhood maltreatment were conducted in peaceful, industrialized European or North American nations. Our knowledge of intergenerational violence in African cultures and regions that have been affected by war remains limited.

Catani (2010) provided a comprehensive review on the effects of traumatized parents on child-rearing practices in war-affected regions: Trauma-related mental disorders often affect the well-being of families and might increase violence against children. Existing studies suggest that the mothers, rather than the traumatized fathers, are responsible for the emotional and physical violence that children in such families experience (Catani, 2010). In a sample of Rwandan mothers, Roth, Neuner, and Elbert (2014) found self-experienced childhood maltreatment to be a more important predictor for abusing and thus traumatizing one's own children than the mothers’ trauma-related symptoms. Hence, in post-conflict settings childhood maltreatment also seems to be a crucial factor for an intergenerational cycle of violence. Recent research suggests that individuals growing up in violent and abusive environments adapt by acquiring predispostions to resort to aggressive behavior within the society (Crombach & Elbert, 2014). Such predispositions, but also the better-known consequences of childhood maltreatment including mental health issues and impaired social functioning, might hamper peace-building efforts in post-conflict countries. Despite the necessity of understanding potential mechanisms and interacting maintaining factors of the intergenerational cycle of violence in war-affected regions, Catani concluded in her review that the number of studies addressing domestic violence is insufficient. In a meta-analytic review of intimate partner violence, Archer (2000) reached the same conclusion when evaluating gender effects.

Research indicates that childhood maltreatment and intimate partner violence are common phenomena in many African societies rooted in traditional patriarchal belief systems (Crombach, Bambonyé, & Elbert, 2014; Roman & Franz, 2013; Straus, 2010; WHO, 2005). Some cross-sectional studies show that the idea of an intergenerational cycle of domestic violence is also applicable to African societies (Abrahams & Jewkes, 2005; Gass, Stein, Williams, & Seedat, 2011; Kishor & Johnson, 2004). The different types of domestic violence tend to be interrelated: (1) experiencing maltreatment during childhood has been identified as a major risk factor for perpetrating violence against the intimate partner (e.g., Abramsky et al., 2011; Ehrensaft et al., 2003; Roberts, McLaughlin, Conron, & Koenen, 2011) and the own offspring (Pears & Capaldi, 2001). (2) Intimate partner violence has been associated with abusive practices against the own children (Guterman & Lee, 2005). Particularly, women experiencing intimate partner violence are more likely to maltreat their children (Taylor, Guterman, Lee, & Rathouz, 2009). Moreover, these women seem to have an elevated risk of engaging in reciprocal intimate partner violence (Archer, 2000; Swan & Snow, 2006). (3) In intimate relationships, the different types of violence, that is, physical violence, sexual violence, and psychological violence, are often used implicitly to exert power and control and cause the partner to experience vulnerability, loss of power and control, and entrapment. Such partner intimidation often occurs almost casually and may happen in a very subtle manner. Nevertheless, it seems to have detrimental effects on health and the quality of the relationships (Coker, Smith, Bethea, King, & McKeown, 2000). Furthermore, initial research suggests that partner intimidation may exacerbate the severity of intimate partner violence (Kelly & Johnson, 2008).

While the effects of childhood maltreatment are most readily apparent at the household level, their ripples can be influenced by broader contextual and historical factors of the country in which the maltreatment occurs. This is evident when examining the case of Burundi, a small and overpopulated country in Eastern Central Africa that has suffered a decade-long conflict between Hutus and Tutsis. In 1993, this conflict escalated into a civil war. The consequences are still evident today in the form of poverty, disrupted families, inheritance-related conflicts, and escalating violence (Human Rights Watch, 2012; United Nations, 2015). Starting a family in these circumstances and having to provide food, shelter, and education for children is a challenging task. Corporal punishment and intimate partner violence are common phenomena and are tacitly accepted at the societal level. These kinds of violence are often described as culturally inherited and/or attributed to difficult living conditions (Sommers, 2013). Based on our experience conducting research and mental health interventions in Burundi, we have the impression that the majority of violence in relationships is committed in a very subtle manner. Consequently, many people and women in particular might feel oppressed or intimidated by their partners without being able to pinpoint the kind of violence that caused these feelings.

Given the prevalence of violence in Burundi, this research investigated experienced partner violence and childhood maltreatment as widely existing problems to be targeted initially if we want to prevent the intergenerational transmission of violence. We anticipated that perpetrating violence against children and romantic partners is also associated with experienced childhood maltreatment in African post-conflict countries. Furthermore, we wanted to explore how gender effects contribute to domestic violence. As part of this objective, we evaluated the impact of experienced partner violence and perceived intimidation in relationships on committing violence against one's own children and romantic partner.

## Methods

### Participants

We invited 141 men and 141 women to participate in the investigation. Their age ranged from 13 to 45 years (mean=27.5 years, SD=7.6 years). The majority of the participants identified as Christian (219, 77.6%), 55 (19.5%) reported being Muslim, and 8 (2.8%) had no religious affiliation. Of the participants, 52 (18.4%) did not have a job, 37 (13.1%) were still going to school, and 18 (6.4%) were students at university. The remaining participants worked as domestic workers, merchants, artisans, teachers, computer scientists, university professors, and medical doctors, for example. However, a significant number of these jobs were not secure employments but rather depending on upcoming opportunities. Overall, 98 (69%) men and 114 (81%) women reported currently living in or having lived in a relationship. Seventy-seven (55%) men and 98 (70%) women had their own children.

### Design and procedure

Between April and June 2013, four psychology students of the University Lumière of Bujumbura conducted interviews in the Bwiza and Buyenzi districts of Bujumbura, the capital of Burundi. These two districts were chosen because the population is very diverse regarding different attributes such as region of origin in Burundi and religious affiliations. Furthermore, Bwiza and Buyenzi are centrally located in Bujumbura. The population is not completely impoverished relative to Burundian standards, while still being comparable to the majority of the poorer districts of the capital. The interviewers visited all streets that constitute the district of Bwiza aiming to approach a similar number of participants on every street. In the district of Buyenzi they approached participants in the same manner. However, of the 25 possible streets, they visited only 7. They chose the participating households at their own discretion while trying to cover the entire street. As many people spent their day outside the interviewers also approached individuals in the streets to identify households within the respective district. Sometimes both individuals of a couple participated. In this case, they were treated as individuals and interviewed separately. Aiming to include working individuals, the interviews were also conducted on weekends. As potential participants, adolescents and adults up to the age of 45 years were accepted because those were most likely to live in active relationships and be involved in the rearing of their own children. Once a household was chosen, interviewers tried to interview between one and three individuals, even if they had to return on the weekends. Only 22 individuals refused to participate, mostly because they were afraid of ongoing governmental enforced redistribution of land and goods at that time and were very mistrustful as a result. The interviewers had received extensive training in collecting data in a cooperation project between the University Lumière of Bujumbura and the University of Konstanz. They conducted the interviews in calm and quiet places, preferably at the home of the participant, and ensured that no other person was present during the conversation. Before starting the investigation, the interviewers informed the participants about the purpose of the study. All participants gave written informed consent to allow their data to be used for scientific publications on condition of anonymity. The legal guardian was also asked to consent for those participants under 18 years of age. The ethical review board of the University Lumière of Bujumbura approved the study.

### Materials

The last author specifically designed all instruments for this investigation. All questions were originally phrased in Kirundi, the local language of Burundi. Regarding socio-demographic information, we asked the participants about age, gender, religious affiliation, current occupation, and relationship status.

We assessed *childhood maltreatment* with five items; both *experienced partner violence* and *perceived partner intimidation* were each assessed with separate 10 items. The possible answers were never (0), sometimes (1), often (2), or very often (3). Total sum scores ranged from 0 to 15 or 0 to 30, respectively. The instruments referred to the experiences throughout the entire lives of the participants. The *childhood maltreatment* items included physical violence, threats, and humiliation by the parents or guardians. Cronbach's α coefficient was 0.78 within a sample of 281 participants (*n*=1 excluded because of missing values). The items loaded statistically significantly onto a single factor accounting for 43% of the total variance. The *experienced partner violence* items included physical violence, sexual violence, threats, humiliations, and forced obedience by the partner. Cronbach's α coefficient of the scale was 0.92 within a sample of 212 participants (*n*=70 excluded because of missing values). The items loaded statistically significantly onto a single factor accounting for 56% of the total variance. The *perceived partner intimidation* items included feeling insecure, threatened, ashamed, imprisoned, scared, controlled, and forced to display a certain behavior because of the partner. Cronbach's α coefficient of the scale was 0.96 within a sample of 201 participants (*n*=81 excluded because of missing values). The items loaded statistically significantly onto a single factor accounting for 72% of the total variance.

We measured violence perpetrated against the children or the partner by asking the participants if they had committed the kinds of violence we had asked them about during the assessment of *experienced partner violence*. The possible answers were yes (1) and no (0) for each question. The total sum score ranged from 0 to 10. Cronbach's α coefficient of the scale assessing *perpetrated violence against children* was 0.61 within a sample of 175 participants (*n*=107 excluded because of missing values). Items loaded statistically significantly onto a single factor, accounting for 30% of the total variance. Cronbach's α coefficient of the scale assessing *perpetrated violence against partner* was 0.65 within a sample of 211 participants (*n*=71 excluded because of missing values). The items loaded statistically significantly onto a single factor accounting for 20% of the total variance.

### Data analysis

As this was the first time, to our knowledge, that intergenerational violence was assessed in Burundi, we chose an explorative approach to identify the relationship between *childhood maltreatment*, *experienced partner violence*, *perceived partner intimidation*, *gender* (0=male, 1=female), and either the perpetration of violence against one's children or romantic partner. We used a backwards stepwise method identifying the best-fitting model using the Akaike Information Criterion. We performed multivariate Poisson regression analyses accounting for the properties of our data.

In our first analysis, we predicted *perpetrated violence against children* with *childhood maltreatment*, *experienced partner violence*, *perceived partner intimidation*, *gender*, and all two-way interactions. We excluded 43 women and 64 men from the analysis because they reported not having children. Due to missing values, we excluded two women. Hence, our sample size for this analysis was *n*=173. In our second analysis, we predicted *perpetrated violence against partner* using the same predictors. We excluded 27 women and 43 men because they had never had a relationship. Due to missing values, we excluded five women and seven men. Hence, our sample size for this analysis was *n*=200. In addition, we calculated Mann–Whitney *U*-tests to confirm the different degree of exposure to risk factors of violent behavior between participants who perpetrated violence and those who did not.

## Results

Means, standard deviations, the range, and the prevalence of the different types of aggression are presented in Table 1 for the samples of the two regression models. We conducted Bonferroni–Holm corrected Mann–Whitney *U*-tests to assess gender differences. In both samples, the tests revealed that women reported higher degrees of *experienced partner violence* and *perceived partner intimidation* than men (all *p*'s<0.001). Men reported higher levels of *perpetrated violence against partner* than women (all *p*'s<0.001).

**Table 1:** Predicting and dependent variables of the two regression models

	First sample (*n*=173)			Second sample (*n*=200)		
		
	Men (*n*=77)	Women (*n*=96)	Total (*n*=173)	Men (*n*=91)	Women (*n*=109)	Total (*n*=200)
Childhood maltreatment, mean (SD) [range] ∣% of prevalence∣	6.1 (3.4) [0–13] ∣95∣	5.5 (3.8) [0–15] ∣93∣	5.8 (3.7) [0–15] ∣94∣	6.2 (3.4) [0–13] ∣95∣	5.3 (3.7) [0–15] ∣94∣	5.7 (3.6) [0–15] ∣94∣
Experienced partner violence, mean (SD) [range] ∣% of prevalence∣	1.7 (2.8) [0–12] ∣51∣	8.3 (7.6) [0–27] ∣91∣	5.4 (6.8) [0–27] ∣73∣	1.9 (2.7) [0–12] ∣65∣	7.6 (7.5) [0–27] ∣87∣	5.0 (6.5) [0–27] ∣73∣
Perceived partner intimidation mean (SD) [range] ∣% of prevalence∣	2.1 (3.5) [0–19] ∣47∣	10.6 (9.8) [0–30] ∣82∣	6.8 (8.7) [0–30] ∣67∣	2.3 (3.4) [0–19] ∣52∣	9.9 (9.9) [0–30] ∣77∣	6.5 (8.5) [0–30] ∣66∣
Perpetrated violence against children mean (SD) [range] ∣% of prevalence∣	1.2 (1.2) [0–4] ∣61∣	1.5 (1.5) [0–5] ∣66∣	1.4 (1.4) [0–5] ∣64∣	–	–	–
Perpetrated violence against partner mean (SD) [range] ∣% of prevalence∣	2.7 (2.0) [0–8] ∣87∣	1.2 (1.1) [0–5] ∣68∣	1.8 (1.7) [0–8] ∣76∣	2.7 (1.9) [0–8] ∣88∣	1.1 (1.1) [0–5] ∣65∣	1.8 (1.7) [0–8] ∣75∣

“–” indicates that statistics correspond to the statistics of the second sample.

**Fig. 1:**
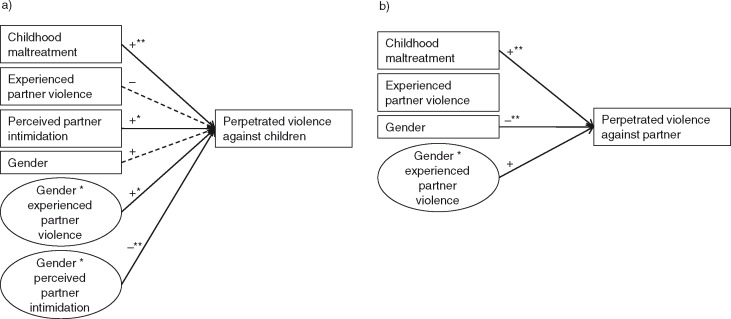
Predictors of violence perpetrated against (a) children and (b) romantic partners.+ and − indicate positive and negative correlations. Dotted lines (*p*≤0.1), solid lines (*p*≤0.05), one asterisk (*p≤0.01), and two asterisks (**p≤0.001) indicate the level of significance.

The final model predicting the number of types of *perpetrated violence against children* is presented in Fig. 1a. We indicated the level of significance and the direction of the relationship, because the β's cannot be interpreted in a linear way and might therefore be misleading (Gagnon, Doron-LaMarca, Bell, O'Farrell, & Taft, 2008). Strongly associated with *perpetrated violence against children* were *childhood maltreatment* (β=0.11, β_ SE_=0.02, Wald-$\chi_{\mathrm{(1)}}^{2}$=32.05, *p*≤0.001), *perceived partner intimidation* (β=0.07, β_SE_=0.03, Wald-$\chi_{\mathrm{(1)}}^{2}$=7.06, *p*=0.008), the interaction between *gender* and *perceived partner intimidation* (β=−0.11, β_SE_=0.03, Wald-$\chi_{\mathrm{(1)}}^{2}$=13.92, *p*≤0.001), and the interaction between *gender* and *experienced partner violence* (β=0.11, β_SE_=0.04, Wald-$\chi_{\mathrm{(1)}}^{2}$=8.19, *p*=0.004). *Gender* (β=0.41, β_SE_=0.22, Wald-$\chi_{\mathrm{(1)}}^{2}$=3.55, *p*=0.059) and *experienced partner violence* (β=−0.07, β_SE_=0.04, Wald-$\chi_{\mathrm{(1)}}^{2}$=3.43, *p*=0.064) were significant in tendency. The significant likelihood ratio test ($\chi_{\mathrm{(6)}}^{2}$=49.06, *p*≤0.001) indicates a good model fit. Pearson's χ^2^/df=1.20 indicates that overdispersion does not affect the model (Gagnon et al., 2008).

The final model predicting *perpetrated violence against partner* is presented in Fig. 1b. Strongly associated with the *perpetrated violence against partner* were *childhood maltreatment* (β=0.09, β_SE_=0.02, Wald-$\chi_{\mathrm{(1)}}^{2}$=31.27, *p*≤0.001), *gender* (β=−1.34, β_SE_=0.16, Wald-$\chi_{\mathrm{(1)}}^{2}$=68.91, *p*≤0.001), and the interaction between *gender* and *experienced partner violence* (β=0.05, β_SE_=0.02, Wald-$\chi_{\mathrm{(1)}}^{2}$=4.40, *p*=0.036). *Experienced partner violence* (β=−0.001, β_SE_=0.02, Wald-$\chi_{\mathrm{(1)}}^{2}$=0.003, *p*=0.96) was not significant. The significant likelihood ratio test ($\chi_{\mathrm{(4)}}^{2}$=136.01, *p*≤0.001) indicates a good model fit. Pearson's χ^2^/df=0.92 indicates that overdispersion does not affect the model (Gagnon et al., 2008).

Only 6% of the participants in both subsamples (11 within the subsample predicting *perpetrated violence against children* and 12 within the subsample predicting *perpetrated violence against the partner*) reported having not experienced any kind of *childhood maltreatment*. Of those who had experienced *childhood maltreatment*, 65% reported having used violence against their children and 76% reported having used violence against their intimate partner. Hence, 35% and 24% of the participants did not perpetrate violence against their children or their partner, respectively, despite the fact that they had been maltreated throughout their childhood. Reflecting the complexity of aggressive behavior and in line with our regression analyses, those participants were less exposed to the other risk factors predicting violent behavior. The participants who reported not using violence against their children were less exposed to partner violence and partner intimidation (all *p*'s<0.033). The participants not engaging in intimate partner violence despite their maltreatment experience were significantly less exposed to partner violence (*p*≤0.021).

## Discussion

Consistent with previous studies (e.g., Markowitz, 2001; Saile, Ertl, Neuner, & Catani, 2014), we found that childhood maltreatment and female guardian victimization by their romantic partner were strong predictors for the perpetration of violence against children. Furthermore, the effects of feeling intimidated by the romantic partner on perpetration of violence toward children were gender-specific: men became more aggressive toward their children, while women became less aggressive. Possibly, men react violently against their children when feeling intimidated by their partner because they understand aggression as a means of reasserting their threatened manhood (Weaver, Vandello, Bosson, & Burnaford, 2010). Consistent with this interpretation and findings suggesting that intimate partner violence perpetrated by women is often trivialized (Archer, 2000), we found that experienced partner violence reduced the likelihood of men acting out against their own children. Hence, the perceived intimidation and not the actual amount of experienced violence by the romantic partner increased the likelihood of men lashing out against their children. Overall, women used slightly more violence toward their children than did men, possibly because they are more involved in child-rearing.

Identifying childhood maltreatment as a strong predictor for intimate partner violence confirms the existence of this association in African post-conflict countries (Kwong et al., 2003). The relatively strong gender effect indicates that men are more likely to use violence against their partner in such societies. This result adds weight to the assumption that “men's physical aggression toward their partners may be much greater, and women's may be greatly curtailed, where traditions inhibiting men from hitting women are absent and where patriarchal values are foremost” (Archer, 2000, p. 667). Another explanation might be the greater involvement of men in war, enhancing trauma-related mental disorders and thereby male intimate partner violence (Sherman, Sautter, Jackson, Lyons, & Han, 2006). Interacting with childhood maltreatment, war-related traumatic/violent experiences contribute to a cycle of violence by exacerbating the potential risk factors of violent behavior (Nandi, Crombach, Bambonyé, Elbert, & Weierstall, 2015). As women were more likely to engage in intimate partner violence when being victimized, they most likely did so defending themselves.

Even though childhood maltreatment is an important predictor for perpetrating domestic violence, our analysis reflects the necessity of understanding violent behavior as a result of several interacting factors. An alarming rate of 94% of the participants reported having experienced physical and/or emotional childhood maltreatment. However, those less exposed to partner violence and partner intimidation were less likely to perpetrate domestic violence. This finding discloses potential starting points to disrupt the intergenerational transmission of violence. Further starting points could be cultural beliefs about child rearing and partnerships, hazardous drinking behavior, exposure to traumatic experiences, and trauma-related mental disorders (Gage, 2005; Saile et al., 2014; Saile, Neuner, Ertl, & Catani, 2013).

The study has several limitations. We relied on self-reports provided by only one generation. Hence, the data might be biased by individual interpretations, memory effects, and by the fact that admitting aggressive behavior is a social taboo. Particularly, reporting sexual aggression against children or very severe punishment seems unlikely for these reasons. As this study was intended as a pilot investigation, we did not assess several factors that might also have an impact on the perpetration of violence against one's children or intimate partner: We did not assess in detail the participants’ economic situation, their education level, or the age and exact number of their children. Nor did we assess the exposure to war-related and other traumatic experiences in detail, and did not screen for mental disorders, even though they are important for understanding the intergenerational cycle of violence. Last but not the least our sample is not representative for the entire population in Burundi. We chose two districts of Bujumbura where people with a variety of cultural and religious attributes of the Burundian society are living. However, this sample most likely differs from the rural population regarding occupation and socio-economic status.

Nevertheless, this study highlights that violence is transferred via behavioral patterns from one generation to the next and that this phenomenon is also applicable to African societies. We identified childhood maltreatment as the strongest and most significant factor in cultivating intergenerational aggression. Our results, particularly combined with those of studies showing the negative impact of childhood violence on school progress, highlight the necessity of addressing childhood maltreatment instead of hiding it behind cultural norms (Crombach et al., 2014). Protecting children against violence will have long-lasting effects on societies, reducing domestic violence, mental health problems, and thereby rendering societies more functional.

## Supplementary Material

Intergenerational violence in Burundi: Experienced childhood maltreatment increases the risk of abusive child rearing and intimate partner violence
