# Supplementary material for: Intergenerational violence in Burundi: Experienced childhood maltreatment increases the risk of abusive child rearing and intimate partner violence
Source: Eur J Psychotraumatol. 2015 Dec 15;6:10.3402/ejpt.v6.26995. doi: 10.3402/ejpt.v6.26995 (PMC4696461; doi:10.3402/ejpt.v6.26995)
Supplement: Intergenerational violence in Burundi: Experienced childhood maltreatment increases the risk of abusive child rearing and intimate partner violence [file EJPT-6-26995-s001.pdf]

Intergenerational violence in Burundi: Experienced childhood maltreatment increases the risk of abusive child rearing and intimate partner violence

Anselm Crombach[1, 2, 3] & Manassé Bambonyé[2]

Incamake

Ivyiyumviro nshingiro: Gukorerwa amabi canke gutotezwa mu buto biragira ingaruka mbi ku magara yo mu mutwe mu buzima bwose bw'abantu vyashikiye kandi bigashobora gutuma uguhohoterana bishobora gukurikirana hagati y'urunganwe n'urundi. Ugutahura inkurukizi mbi z'uguturubikwa bijanye no kurera nabi abana hamwe n'uguhohoterana hagati y'abubakanye birashobora kugira akamaro ntangere mu gutorera inyishu izo nkurikizi mbi zijanye n'amatati hagati y'imibano muri Afrika.

Intumbero: Dufatiye akarorero ku Burundi, dufise umwitwarariko wo guca hirya n'ino ingene uguturubikwa mu buto, guhohoterana hagati y'abubakanye, guterwa ubwoba n'uwo mwubakanye, hamwe n'urugero umuntu azokubita umwana wiwe canke uwo bakundanye.

Ubuhinga: Twarondeye abagabo 141 n'abagore 141 baba k'umugwa mukuru wa Bujumbura. Twaratoyemwo abafise abana babo, n'ababanye hamwe bakundana canke bigeze kubana hamwe bakundana. Dukoresheje urutonde rw'ibibazo rutumenyesha abaturubitswe, abakorewe amabi n'abahohotewe mu buto bwabo hamwe ningene bijanye no gukora ayo mabi canke uko guhohoterana.

Ivyavuyemwo: Twasanze guturubikwa mu buto n'ugutera ubwoba uwo mwubakanye arivyo vyerekana cane abantu bazobikorera abana babo muri kazoza. Vyongeye,twasanze abagore bakorewe amabi canke bahohotewe nabo bubakanye aribo bazoyakorera abana babo, mugabo abagore bagiriwe amabi yuguterwa ubwoba gusa ntibazobikorera abana babo. Abagabo nibo bakora amabi hamwe no guhohotera abo bubakanye kurugero runini. Guturubikwa mu buto vyabonetse kandi ko arivyo vyerekana cane abashobora kugirira amabi abandi muri kazoza. Uko abagore bahohoterwa cane nabo bubakanye niko babisuza kubandi.

Icigwa: Uguturubikwa mu buto nico cerekana k'urugero runini uguhohoterana mungo z'abubatse niyo mpamvu bitegerezwa gushimikwako kungirango hahagarikwe urukurikirane rw'ubugizi bwa nabi nkinkomoko zintambara.

Citation: European Journal of Psychotraumatology 2015, **6**: 26995 - http://dx.doi.org/10.3402/ejpt.v6.26995
